# Frontal lobe development in fetuses with growth restriction by using ultrasound: a case–control study

**DOI:** 10.1186/s12884-022-05126-8

**Published:** 2022-11-21

**Authors:** Ruan Peng, Qiao Zheng, Li-Hong Wu, Xia Yin, Ju Zheng, Hong-Ning Xie

**Affiliations:** Department of Ultrasonic Medicine, Fetal Medical Centre, The First Affiliated Hospital of Sun Yat-Sen University, Zhongshan 2nd Road 58#, Guangzhou, Guangdong China

**Keywords:** Fetal circulatory redistribution, Fetal growth restriction, Frontal anteroposterior diameter, Frontal lobe growth, Prenatal ultrasonography

## Abstract

**Background:**

Fetal growth restriction (FGR) occurs in up to 10% of pregnancies and is a leading cause of perinatal mortality and neonatal morbidity. Three-dimensional ultrasonography of intracranial structure volume revealed significant differences between fetuses with FGR and appropriate for gestational age (AGA) fetuses. We aimed to compare the frontal lobe development between fetuses with FGR and appropriately grown fetuses and evaluate the impact of fetal circulatory redistribution (FCR) on frontal lobe development in fetuses with FGR.

**Methods:**

We performed a case–control study at our institution from August 2020 to April 2021. The frontal antero-posterior diameter (FAPD) and occipito-frontal diameter (OFD) were measured on the trans-ventricle view and we calculated the Z-scores for FAPD and OFD standardized for gestational age (GA) and transverse cerebellar diameter (TCD) by performing a standard regression analysis followed by weighted regression of absolute residual values in appropriately grown fetuses. We calculated the FAPD/OFD ratio as 100 × FAPD/OFD and FAPD/HC (head circumference) as 100 × FAPD/HC. To compare intracranial parameters, we randomly selected a control group of appropriately grown fetuses matched with the FGR group at the time of ultrasonography. We performed between-group comparisons of the FAPD Z-score, OFD Z-score, FAPD/OFD ratio and FAPD/HC. Similarly, we compared intracranial parameters between fetuses with FGR with and without FCR.

**Results:**

FAPD/OFD ratio was curvilinear related to all the independent variables (GA, BPD, FL, and TCD). Compared with appropriately grown fetuses, fetuses with FGR showed a significantly lower FAPD/OFD ratio, FAPD Z-score, and FAPD/HC. There was no significant difference in the FAPD Z-score, FAPD/OFD ratio, and FAPD/HC between FGR fetuses with and without FCR.

**Conclusions:**

The FAPD/OFD ratio varied during pregnancy, with a mild reduction before and a mild increase after about 33 gestational weeks. Fetuses with FGR showed reduced frontal lobe growth; moreover, fetal frontal lobe development disorders were not significantly different in fetuses with FCR.

**Trial registration:**

Date: 09–27-2017; Number: [2017]239.

## Background

Fetal growth restriction (FGR) occurs in up to 10% of pregnancies [1]. The ISUOG-FGR definition follows the Delphi consensus criteria and includes either estimated fetal weight (EFW) or abdominal circumference (AC) < 3^rd^ percentile or EFW or AC < 10^th^ percentile combined with abnormal Doppler findings or a decrease in growth centiles [2]. FGR is a leading cause of perinatal mortality and neonatal morbidity [3, 4]. It is associated a lower 5-min Apgar scores [5]; and it affects long-term prognosis, including that related to cardiovascular dysfunction, endocrine disease, and neurodevelopmental disorders [6, 7]. FGR is associated with lower neurobehavioral test scores [8, 9], which can be burdensome for patients and their families owing to additional costs, such as expensive caregiving and counselling for mental health.

Previous literatures have demonstrated that three-dimensional ultrasonography of intracranial structure volume revealed significant differences between fetuses with FGR and appropriate for gestational age (AGA) fetuses [10, 11]. However, the limited convenience of three-dimensional ultrasonography has impeded its clinical applications. The frontal antero-posterior diameter (FAPD)/occipito-frontal diameter (OFD) ratio has been recently used to evaluate frontal lobe development in fetuses with congenital heart disease [12]. FAPD was measured from the inner line of the frontal bone to the posterior edge of the CSP along the fetal brain midline, which represents the frontal lobe of the fetal cerebrum. To the best of our knowledge, no study has investigated the usefulness of the FAPD/OFD ratio in FGR assessment. We devised this study to investigate the FAPD/OFD ratio and frontal lobe development in fetuses with FGR. Examining frontal lobe growth in fetuses with FGR may elucidate mechanisms underlying their poor neurodevelopmental outcomes. We thus aimed to compare frontal lobe development between fetuses with FGR and appropriately grown fetuses and evaluate the impact of fetal circulatory redistribution (FCR) on frontal lobe development in fetuses with FGR.

## Methods

### Population selection

We selected all women with singleton pregnancy who attended our hospital and were performed the second and third trimester ultrasound measurements between August 2020 and April 2021. We excluded the fetuses with major congenital abnormalities and loss to follow-up. The gestational age was between 20 and 40^+2^ gestational weeks. FAPD/OFD ratio was measured in all women who visited our institution during the study period. One woman was performed for one measurement. Our institution is a prenatal diagnostic unit and is a referral center. The indication for examination was routine prenatal care or referred for previous history/high-risk population. All participants provided written informed consent. The ethical committee of our institution (ICE for Clinical Research and Animal Trials of the First Affiliated Hospital of Sun Yat-sen University) approved this study. Gestational age (GA) was adjusted to the first day of the last normal menstrual period and confirmed on the first-trimester ultrasonogram at 11 ~ 13^+6^ gestational weeks.

### Measurements

Experienced operators performed biometric measurements, including those for the biparietal diameter (BPD), HC, AC, femur length (FL), humerus length (HL), transverse cerebellar diameter (TCD), and structural evaluation. All fetal biometric and structural assessments were performed following guidelines of the International Society of Ultrasound in Obstetrics and Gynecology [13].

We calculated the estimated fetal weight using BPD, HC, AC, and FL. Fetal weight and weight percentiles were estimated using the Hadlock formula (Hadlock-4) [14] and used Hadlock 1991 for EFW percentiles [15] and Leung 2008 for other biometry percentiles [16]. Birth weight Z-scores were calculated as previously described by Leung, using regression equations [16]. FGR cases were classified, based on gestational age at the time of diagnosis, into early-onset FGR (< 32 weeks) and late-onset FGR (≥ 32 weeks) [17].

**Fig. 1:**
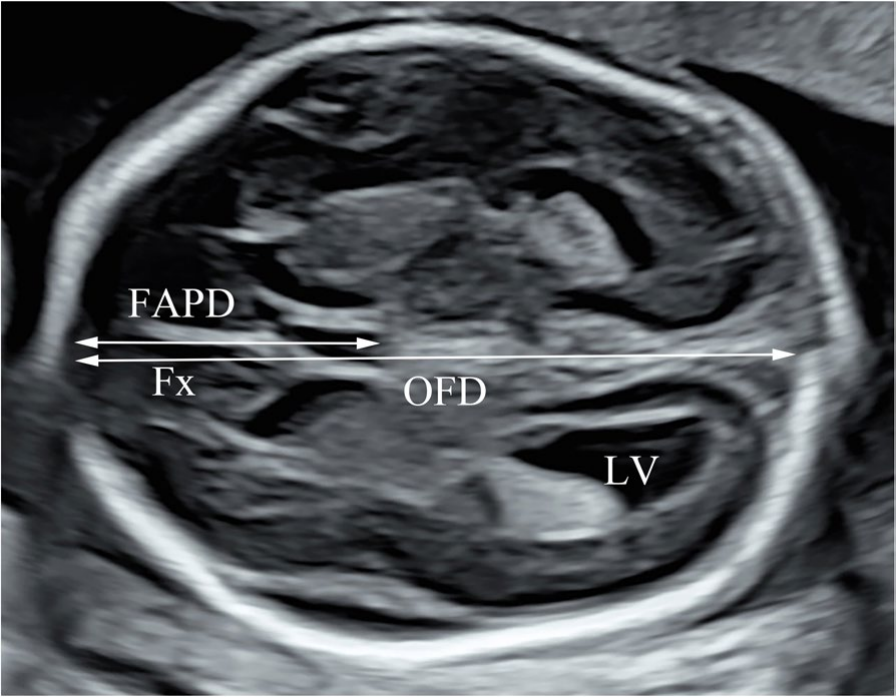
Imaging for fetal brain measurements: measurements of the FAPD and OFD in the trans-ventricle view. FAPD, frontal antero-posterior diameter; OFD, occipito-frontal diameter; Fx, falx; LV, lateral ventricle

Ultrasonography was performed using a Voluson E8 and E10 ultrasonography machine (GE Healthcare, Zipf, Austria), with a RAB2-5 (2 ~ 5 MHz) convex probe. FAPD and OFD were measured retrospectively on the trans-ventricle view by one operator (Fig. 1) [12]. We assumed that the posterior margin of the cavum septum pellucidum (CSP) represents the boundary between the frontal lobe region and the remaining brain regions. FAPD was measured from the inner line of the frontal bone to the posterior edge of the CSP along the fetal brain midline. OFD was the measured distance between the inner edges of the frontal and occipital bones. The FAPD/OFD ratio was calculated as 100 × FAPD/OFD, and FAPD/HC was calculated as 100 × FAPD/HC. These two parameters were used to investigate frontal lobe development with respect to the whole brain. We calculated Z-scores standardized for FL/BPD/GA/TCD, FAPD, and OFD with appropriately grown fetuses. Doppler flow patterns of the middle cerebral artery (MCA), umbilical artery (UA), and left and right uterine arteries (UtA) were acquired during fetal apnea, as previously published [18–20]. We recorded the pulsatility index (PI) for the MCA, UA, and UtA and peak systolic velocity for the MCA. The mean PI for UtA (mUtA) was calculated as (left UtA-PI + right UtA-PI)/2. A mUtA > 95^th^ percentile was considered as increased uterine artery impedance [20]. UA-PI > 95^th^ percentile [19] and/or absent or reversed umbilical artery were considered umbilical artery abnormalities. We calculated the ratio of the MCA to umbilical artery PI as the cerebroplacental ratio (CPR). The CPR was interpreted using GA-related reference ranges [21]. CPR < 5^th^ percentile indicated fetal “brain sparing” [22]. Fetuses with FGR were divided into those with and without FCR based on presence/absence of CPR < 5^th^ percentile. Operators were blinded to fetal biometric measurements and clinical outcomes.

### Group dividing

We divided all fetuses into different groups according to the EFW and AC. FGR was defined by either EFW or AC < 3^rd^ percentile or EFW or AC < 10^th^ percentile combined with abnormal Doppler findings or a decrease in growth centiles or AC below the 10^th^ percentile for GA [2]. To compare intracranial parameters, we randomly included appropriately grown fetuses in a control group matched to the FGR group according to the GA on ultrasonography. We used stratified sampling to derive the random sample. The time window that we allowed for matching was within one week and four controls were matched for one case.

### Clinical data

We collected data regarding fetal biometry, structural ultrasonography findings, and placenta characteristics. We recorded the obstetric history, pre-pregnancy body mass index (BMI), and medical history (gestational diabetes, chronic hypertension, pre-eclampsia, and autoimmune diseases) of mothers. We only obtained genetic test results (fetal karyotyping, microduplications, microdeletions, or whole-exome sequencing) from a minority of our study population. We prospectively collected postnatal data, including GA at birth, birth weight, sex, 5-min Apgar scores, umbilical artery PH, HC, hospitalization length, and neonatal complications. Neonatal complications included respiratory distress syndrome, neonatal sepsis, intraventricular hemorrhage (grade III or IV), cystic periventricular leukomalacia, hypoglycemia (< 2.2 mmol/l), and necrotizing enterocolitis.

### Intra/inter variability

The interobserver variability of FAPD and OFD was calculated by comparing measurements obtained by two independent observers (R.P. and Q.Z.) from data collected from 30 fetuses. Intraobserver variability of FAPD and OFD was calculated by comparing measurements obtained twice by one observer (R.P.) from 30 fetuses. The interobserver 95% limits of agreement were depicted with Bland–Altman plots using MedCalc 9 Software (MedCalc Software, Mariakerke, Belgium). Moreover, we calculated the intraclass correlation coefficient (ICC) to evaluate the interobserver and intraobserver variability. Two-way random models for the ICCs were used.

### Statistics

The analyses were performed with the statistical software packages R 4.0.5 (https://www.r-project.org/, The R Foundation) and IBM SPSS Statistics for Windows version 25.0. Normality tests for FAPD, OFD, FAPD/OFD ratio, and residuals of FAPD and OFD were performed using the Kolmogorov–Smirnov test. Descriptive characteristics were analyzed using means and medians for normally and non-normally distributed data, respectively. We derived the random sample for matching with FGR groups. We calculated the FAPD and OFD Z-scores standardized for GA and TCD by performing standard regression analysis and weighted regression of absolute residual values. We first compared linear, quadratic, cubic, and logarithmic regression models. In case a more complex model, including quadratic, cubic, or logarithmic model, could not improve the correlation coefficient, we used a simpler model, such as a linear model. Further, normal distribution of residuals was checked using Kolmogorov–Smirnov tests. If necessary, the raw data and residuals were transformed, and the new residuals were analyzed. Finally, we examined the residuals and constructed the regression models as independent variables with Altman’s approach [23]. Z-score was calculated as (observed intracranial parameters – predicted intracranial parameters)/predicted standard deviations (SDs).

Differences between the case and control groups and the FGR with and without FCR subgroups were assessed using the chi-square test or Student’s t-test. The SDs or standard errors (SEs) for the variables were reported when necessary. Statistical significance was set at *P* < 0.05. The study size was acquired according to the formula that *n* = [(Z_1-α/2_ × σ)/δ]^2^,α = 0.05, δ = 0.3, σ = 1.0.

## Results

**Fig. 2:**
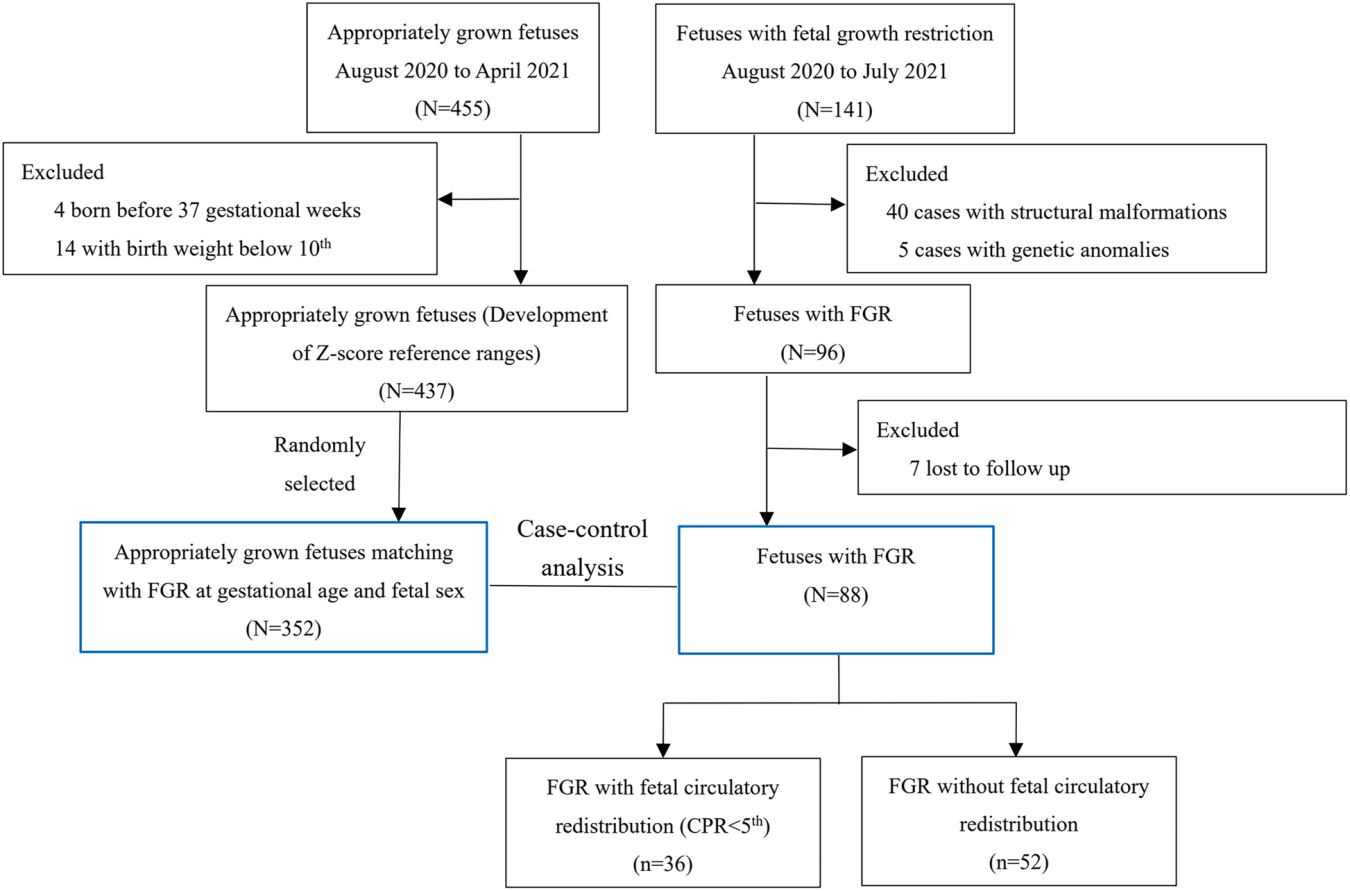
Flow diagram of fetuses with FGR and appropriately grown fetuses. CPR, cerebroplacental ratio; FGR, fetal growth restriction

We included 437 appropriately grown fetuses to establish the Z-score reference ranges for FAPD and OFD. During the study period, 141 fetuses with FGR were identified, among which 48 were referred from other institutions and 93 were identified at 2^nd^ or 3^rd^ trimester screening in our institution. Among 141 fetuses with FGR, 88 met the inclusion criteria and had available outcome data. Finally, the case–control analyses included 88 fetuses with FGR and 352 GA-matched appropriately grown fetuses (Fig. 2). Forty fetuses in FGR and eighteen appropriately grown fetuses were tested for genetic abnormalities. All the neonates were phenotypically normal after birth. Table 1 shows the clinical characteristics of both groups. As shown in Table 1, the groups were matched according to GA on ultrasonography. Additionally, maternal age, pre-pregnancy BMI, and fetal sex were approximately similar between these two groups. Thirty-eight (43.2%) were identified as early-onset FGR and fifty (56.8%) were as late-onset FGR.

**Table 1:** Basic characteristics and neonatal outcomes of fetuses with fetal growth restriction (FGR) and control group

Variables	Control group (*N* = 352)	FGR group (*N* = 88)	*P* value
Basic characteristics			
Gestational age at examination (weeks)	33 (23^+1^ ~ 40^+2^)	33^+1^ (23^+4^ ~ 40^+1^)	0.917
Maternal age (years)	31 (22 ~ 40)	31 (23 ~ 41)	0.590
Pre-pregnancy body mass index (kg/m^2^)	20.2 (15.4 ~ 28.2)	20.3(16.6 ~ 27.4)	0.272
Cigarette smoking in pregnancy	0 (0%)	1 (1.14%)	-
Maternal disease	0 (0%)	21 (23.9%)	< 0.001
Nulliparous	233 (66.2%)	61 (69.3%)	0.578
Ultrasound examination			
mUtA	0.70 (0.35 ~ 1.83)	0.89 (0.40 ~ 1.79)	0.525
Cephalic presentation	275 (78.1%)	74 (84.1%)	0.216
EFWp	52.5% (99 ~ 12.2%)	3% (8.1 ~ < 1%)	< 0.001
UmA-PI	1.01 (0.53 ~ 1.46)	1.71 (0.68 ~ 1.81)	0.006
MCA-PI	1.97 (0.93 ~ 3.98)	1.68 (0.93 ~ 2.57)	0.004
Delivery and neonate			
Gestational age at birth (weeks)	39^+1^ (37 ~ 41)	37 (27^+1^ ~ 40^+4^)	< 0.001
Cesarean at delivery	127 (36.1%)	44 (50.0%)	0.017
Birth weight (g)	3240 (2500 ~ 3950)	2300 (780 ~ 2880)	< 0.001
Birth weight Z-score	0.38 (-1.19 ~ 1.99)	-2.15 (-5.16 ~ -1.34)	< 0.001
Neonatal head circumference (cm)	34 (32 ~ 36)	32 (24 ~ 34)	< 0.001
Neonatal head circumference Z-score	0.85 (-0.99 ~ 3.75)	-0.34 (-3.88 ~ 0.98)	< 0.001
Male	201 (57.1%)	57 (64.8%)	0.191
5-min Apgar score ≤ 7	14 (4.0%)	11 (12.5%)	0.002
Lower umbilical artery PH (≤ 7.20)	9 (3.2%) ^a^	18 (20.5%) ^a^	< 0.001
Length of hospitalization (days)	3 (2 ~ 12)	6 (2 ~ 66)	0.006
Neonatal complications	14 (4.0%)	11 (12.5%)	0.002

Data are presented as n (%) or median (range)

*PI* Pulsatility index, *mUtA* Mean PI of uterine artery, *EFWp* Percentiles of EFW, *UmA* Umbilical artery, *MCA* Middle cerebral artery

^a^ Umbilical artery PH was obtained from 283 appropriately grown fetuses and 68 fetuses with FGR. Maternal disease includes gestational diabetes, chronic hypertension, pre-eclampsia, and autoimmune diseases

The FAPD, OFD, and FAPD/OFD ratios were normally distributed (*P* = 0.093,* P* = 0.120, and* P* = 0.185, respectively) in 437 appropriately grown fetuses, with means of 34.7, 84.5, and 40.92, respectively. Comparisons among different regression models of the FAPD and OFD based on the independent variables (GA and TCD) are shown in Table 2.

**Table 2:** Comparisons among different regression models of the fetal frontal antero-posterior diameter and occipito-frontal diameter based on the gestational age and transverse cerebellar diameter in appropriately grown fetuses

Parameters	R^2^				
	Linear regression model	Quadratic regression model	Cubic regression model		Logarithmic regression model
Gestational age (weeks)					
FAPD	0.920	0.939	0.939	0.936	
OFD	0.931	0.958	0.958	0.951	
Transverse cerebellar diameter (mm)					
FAPD	0.879	0.926	0.927		0.917
OFD	0.883	0.943	0.944		0.929

None of the correlation coefficients were adjusted regression coefficients

*FAPD*: Frontal antero-posterior diameter, *OFD* Occipito-frontal diameter

**Fig. 3:**
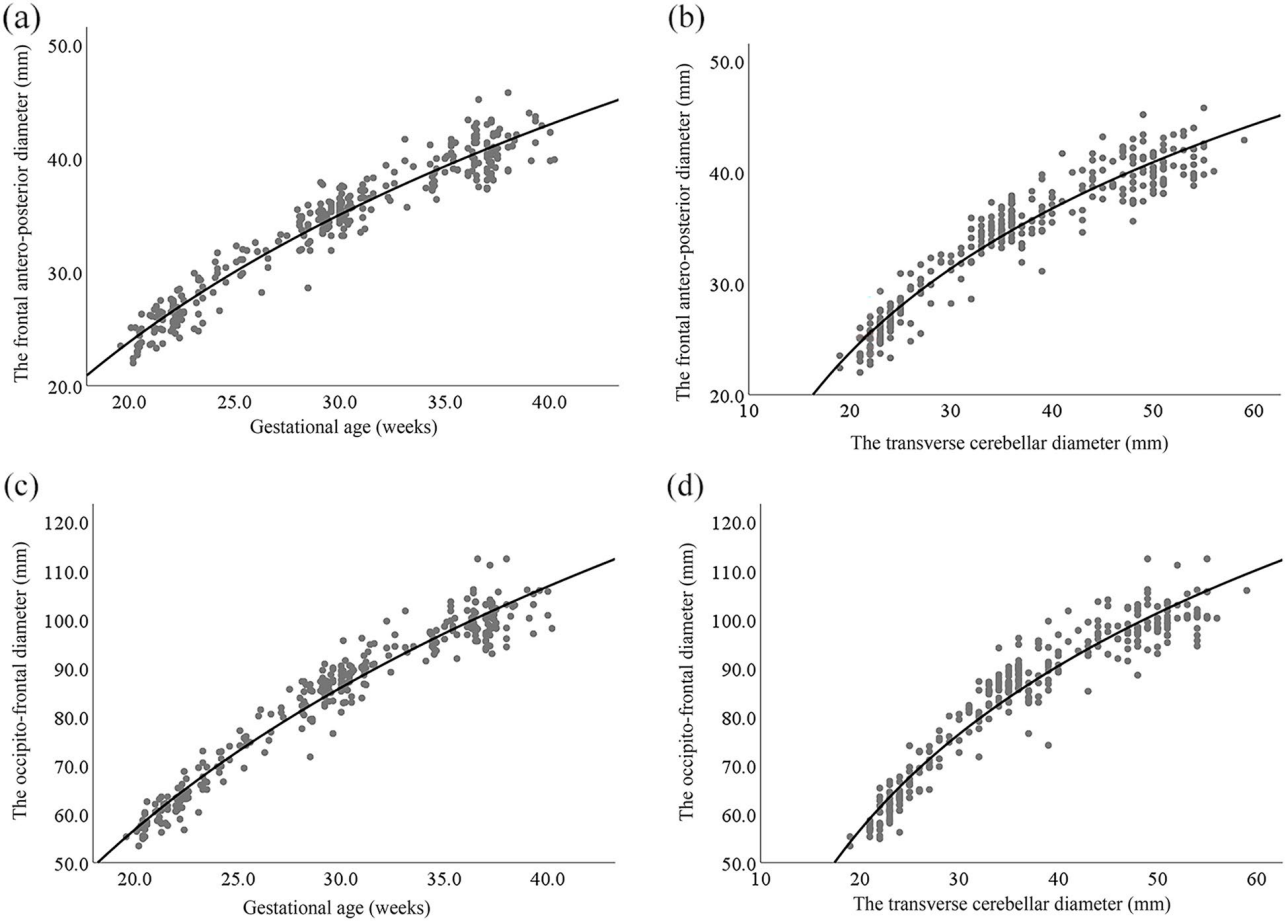
The logarithmic relationship between gestational age, and transverse cerebellar diameter and frontal antero-posterior diameter (**a** and **b**) and occipito-frontal diameter (**c** and **d**) based on measurements of 437 appropriately grown fetuses

A cubic regression model was the best model for describing the relationship among the independent variables (GA and TCD) and FAPD and OFD. However, the residuals were non-normally distributed for the cubic regression models. Therefore, we chose a logarithmic regression model for establishing the Z-score reference ranges for the FAPD and OFD. There were clear relationships of TCD with FAPD and OFD in appropriately grown fetuses (*R*^2^ = 0.917, *P* < 0.001 and *R*^2^ = 0.929, *P* < 0.001, respectively) (Fig. 3). Table 3 shows logarithmic regression models of FAPD and OFD according to the independent variables (GA and TCD). The distribution of residuals was normal and bell-shaped. Normal distribution of residuals is necessary for establishing the Z-score reference ranges of the FAPD and OFD.

**Table 3:** Logarithmic regression models for predicting the fetal frontal antero-posterior diameter and occipito-frontal diameter based on the gestational age and transverse cerebellar diameter in appropriately grown fetuses (the regression equations for mean are described as y = a + b Ln (x) and the regression equations for standard deviations are described as y’ = a’ + b’ Ln (x’))

Parameters	Formula for											
	Estimated Mean						Estimated SDs					
	a			b			a’			b’		
	Value	SEs	*P*	Value	SEs	*P*	Value	SEs	*P*	Value	SEs	*P*
Gestational age (GA) (weeks)												
FAPD	-59.133	1.366	< 0.001	27.689	0.404	< 0.001	-1.138	0.794	0.153	0.693	0.235	0.003
OFD	-159.380	3.085	< 0.001	72.136	0.913	< 0.001	-5.034	1.949	0.010	2.251	0.577	< 0.001
Transverse cerebellar diameter (TCD) (mm)												
FAPD	-32.306	1.117	< 0.001	18.718	0.313	< 0.001	-1.073	0.665	0.108	0.678	0.186	< 0.001
OFD	-89.142	2.685	< 0.001	48.666	0.752	< 0.001	-2.235	1.605	0.165	1.532	0.450	0.001

*BPD* Biparietal diameter, *FAPD* Frontal antero-posterior diameter, *OFD* Occipito-frontal diameter, *TCD* Transverse cerebellar diameter, *SEs* Standard errors

**Fig. 4:**
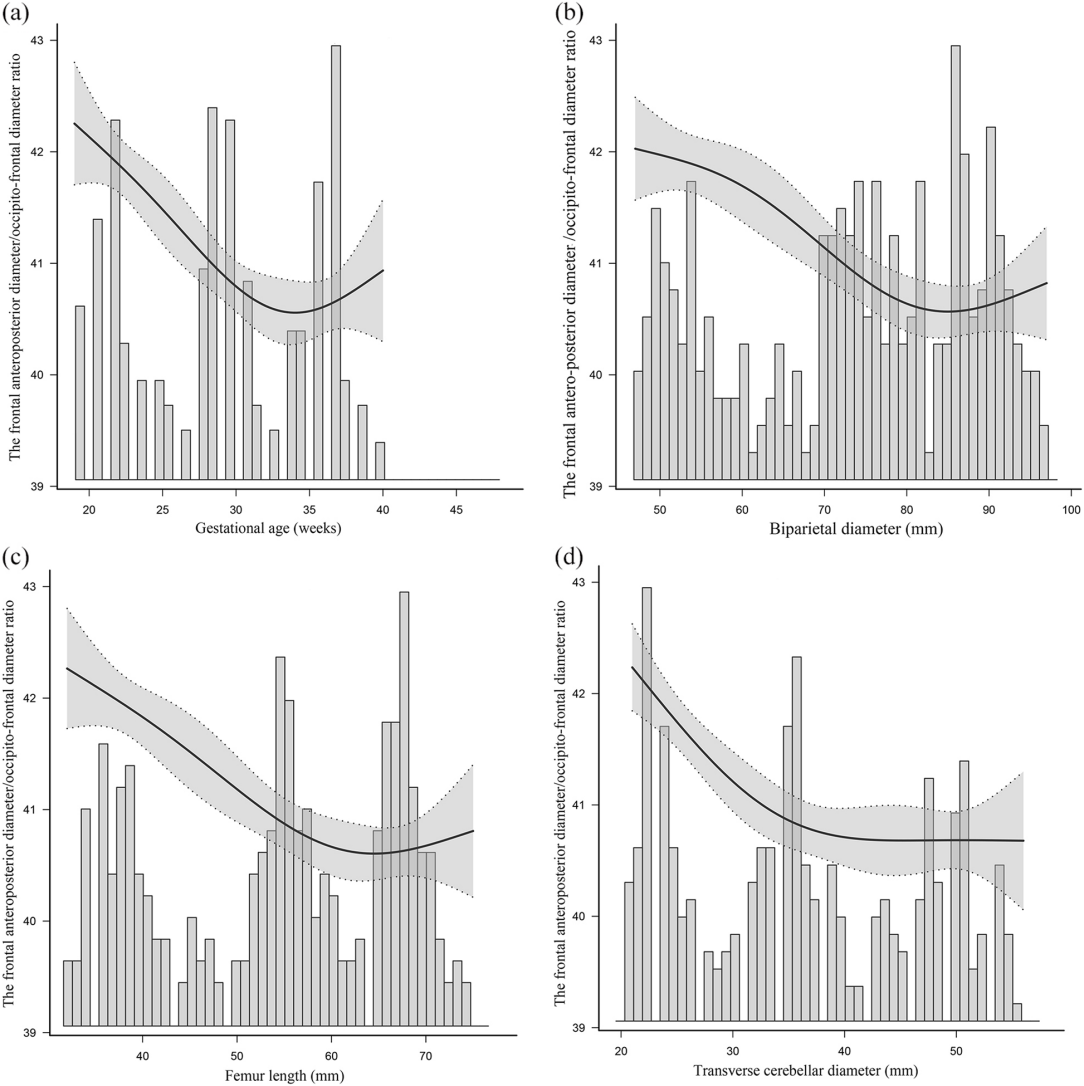
There was a curvilinear relationship among gestational age (GA) **a** biparietal diameter (BPD) **b** femur length (FL) **c** transverse cerebellar diameter (TCD) **d** and the frontal antero-posterior diameter (FAPD)/occipito-frontal diameter (OFD) ratio based on measurements of 437 appropriately grown fetuses. The bars represent the results of FAPD/OFD ratio in different gestation ages or different BPD / FL / TCD values. Mean (**——**) of FAPD/OFD ratio in the 437 appropriately grown fetuses and 95% confidence intervals (……) of the FAPD/OFD ratio are shown

The mean FAPD/OFD ratio was 40.92 ± 1.25 in 437 appropriately grown fetuses. There was a curvilinear association between the FAPD/OFD ratio and all independent variables (GA, BPD, FL, and TCD) (Fig. 4). There were variations in the FAPD/OFD ratio during fetal growth, with a mild reduction with increasing GA before about 33 gestational weeks and a mild increase after about 33 gestational weeks. The changing trends could be seen in Fig. 4. The FAPD/OFD ratio illustrated a likely “U” shape variation during pregnancy.

The FAPD and OFD Z-scores standardized for TCD were used for comparisons because the volume of the cerebellum is not influenced by impaired fetal growth.^10^ The mean FAPD Z-score was significantly lower in fetuses with FGR (-2.55 ± 1.48) than in appropriately grown fetuses (0.17 ± 1.20) (*P* < 0.001). The mean FAPD/OFD ratio was significantly lower in the FGR cohort (37.33 ± 1.31) than in the control cohort (40.62 ± 1.00) (*P* < 0.001). Table 4 presents the between-group comparisons of intracranial parameters and the FAPD and OFD Z-scores were calculated using a model with cerebellum diameter.

**Table 4:** Comparisons of intracranial parameters between the control and fetal growth restriction groups

Parameters	Control group (*N* = 352)	FGR group (*N* = 88)	*P*
FAPD (mm)	37.20 ± 3.81	32.28 ± 3.44	< 0.001
FAPD Z-score	0.17 ± 1.20	-2.55 ± 1.48	< 0.001
OFD (mm)	91.59 ± 9.41	87.02 ± 9.96	< 0.001
OFD Z-score	0.21 ± 1.22	-0.38 ± 1.45	0.003
FAPD/OFD ratio	40.62 ± 1.00	37.33 ± 1.31	< 0.001
FAPD/HC	12.81 ± 0.60	11.80 ± 0.68	< 0.001

Data given as mean ± standard deviation. The FAPD and OFD Z-scores were calculated using a model with cerebellum diameter

*FGR* Fetal growth restriction, *FAPD* Frontal antero-posterior diameter, *OFD* Occipito-frontal diameter, *HC* Head circumference

There was no significant difference in the distribution of FAPD/OFD ratios between fetuses with FGR with and without FCR (37.56 ± 1.16 and 37.20 ± 1.40, respectively; *P* = 0.383). The FAPD/HC was not significantly different between fetuses with FGR with and without FCR (11.92 ± 0.63 and 11.77 ± 0.72, respectively; *P* = 0.410). Table 5 demonstrates the between-subgroup comparisons of intracranial parameters. Furthermore, there was no significant difference in FAPD/OFD ratio between fetuses with early-onset and late-onset FGR (37.58 ± 1.39 and 37.00 ± 1.15, respectively*; P* = 0.116).

**Table 5:** Comparisons of intracranial parameters between fetal growth restriction cases with and without FCR

Parameters	With FCR (*n* = 36)	Without FCR (*n* = 52)	*P*
FAPD Z-score	-2.50 ± 1.45	-2.56 ± 1.62	0.935
OFD Z-score	-0.40 ± 1.29	-0.37 ± 1.60	0.902
FAPD/OFD ratio	37.56 ± 1.16	37.20 ± 1.40	0.383
FAPD/HC	11.92 ± 0.63	11.77 ± 0.72	0.410

Data given as mean ± standard deviation. The FAPD and OFD Z-scores were calculated using a model with cerebellum diameter

*FCR* Fetal circulatory redistribution, *FAPD* Frontal antero-posterior diameter, *OFD* Occipito-frontal diameter, *HC* Head circumference

The intra- and interobserver ICCs for the FAPD and OFD were > 0.95, indicating good reliability. Table 6 shows the interobserver agreement and intra/interobserver reliability for ultrasonographic measurements of FAPD and OFD. Bland–Altman plots show the interobserver agreement measurements of FAPD and OFD in 30 appropriately grown fetuses.

**Table 6:** Interobserver agreement and intra/interobserver reliability for measuring the frontal antero-posterior diameter (FAPD) and occipito-frontal diameter (OFD) with ultrasonography

Parameters	FAPD	OFD
Interobserver agreement		
Mean difference 95% CI (mm)	0.06 (-0.55 ~ 0.42)	-0.40 (-1.37 ~ 0.58)
95% LOA (mm)	-2.60 ~ 1.64	-5.50 ~ 3.03
Intraobserver ICC (95% CI)	0.98 (0.97 ~ 0.99)	0.98 (0.97 ~ 0.99)
Interobserver ICC (95% CI)	0.97 (0.95 ~ 0.99)	0.98 (0.97 ~ 0.99)

*CI* Confidence interval, *LOA* Limits of agreement, *ICC* Intraclass correlation coefficient

## Discussion

The FAPD/OFD ratio varied during pregnancy, with a mild reduction before and a mild increase after about 33 gestational weeks. FGR showed reduced frontal lobe growth. Moreover, based on a comparison of FGR fetuses with and without FCR, FCR may not be significantly associated with development disorders of the fetal frontal lobe.

Infants born at term or preterm with birth weights lower than the AGA values are at higher risk of developmental impairments than those with birth weights AGA [24]. Neurodevelopmental impairments vary across different domains in infants with FGR. Children with FGR show specific impairments in the cognitive performance, behavior, and hearing development domains [25]. FGR-related neurodevelopmental disorders were previously evaluated using the HC, with a small fetal head size being considered most effective for predicting adverse neurodevelopment in fetuses with FGR [26, 27]. However, reduced HC could not explain variations in neurodevelopmental disorders across domains. This study showed that fetuses with FGR show reduced frontal lobe development, which is not influenced by FCR. The study findings may elucidate mechanisms underlying their poor neurodevelopmental outcomes.

Cognitive and academic deficits occur in children with FGR aged 6–8 years, even when born at term [28]. Early insults to the intrauterine environment, including chronic hypoxia and malnutrition, may disturb neural architecture development in the frontal lobe, which is crucial for executive function development after birth. Furthermore, the fetal frontal lobe is especially vulnerable to nutrient deficiency in the third trimester than other areas [29]. Our findings confirmed impaired development of the fetal frontal lobe in fetuses with FGR, which could explain the abnormal cognitive performance, behavior, and hearing development in childhood, including that at school age. Benavides–Serralde reported significantly reduced frontal lobe volume in severe intrauterine growth restriction (IUGR) cases using three-dimensional ultrasonography, even after adjustment for the BPD [10]. Imad R demonstrated that frontal lobe and HC are similarly reduced in FGR [30]. In this study, we also found that FGR showed reduced frontal lobe growth and reduced HC. Additionally, reduced frontal lobe growth was more obvious than HC. Impaired frontal lobe development is also noted in fetuses with congenital heart defect [12] and Down syndrome [31].

Examining variation patterns in the FAPD and FAPD/OFD ratio can reveal changes in the fetal frontal lobe during pregnancy. We found that the FAPD/OFD ratio is relatively fixed; however, it showed a mild variation during fetal development. These changes suggest that growth velocities differ across different brain regions. Myelination begins from the 3^rd^ or 6^th^ month of fetal life. Myelination sequentially proceeds from the internal capsule to the occipital, splenium, parietal, body of corpus callosum, temporal, frontal, and genu white matter regions [32, 33]. Variations in the FAPD/OFD ratio may be caused by this mechanism.

We observed a non-significant reduction in brain hemodynamic redistribution in the frontal lobe region in FGR fetuses with FCR when compared with those without FCR. There was no difference in the FAPD Z-score and FAPD/OFD ratio between fetuses with FGR with and without FCR. We speculate that in the FGR fetuses with FCR, the frontal lobe growth is reduced but it is not more severe than without FCR. However, a systematic review published in 2014 reported that children with FGR with FCR had more severe neurodevelopmental impairment than those without FCR [25]. For fetuses with FGR combined with FCR (cardiac output redistribution toward the myocardium and brain), the timing and magnitude of these vascular redistribution and nutrient delivery changes are associated with the fetal growth pattern. Fetuses with IUGR at the early deterioration stage show an overall increase in blood flow perfusion, which is mainly manifested in the frontal lobe. Additionally, the findings of reduced FAPD/OFD ratio in fetuses with FGR should be interpreted with caution. There remained a small degree of overlap between the two studied populations.

Although the delivery timing is a potential modifier of the association between FGR and neurodevelopmental impairment [33, 34], we did not compare intracranial structures between term and preterm cases. Instead, we investigated fetal frontal lobe development, which is not associated with the time of delivery. In our study, frontal lobe size reduction is not significantly different between early and late FGR, which may be caused by the fact that the case number is relatively small in our cohort.

Our study has several strengths. We focused on a clinically important population, namely fetuses with growth restriction with poor neurodevelopmental prognosis. The FAPD/OFD ratio, as a predictor, is flexible and convenient and can be easily obtained on the trans-ventricle view during BPD and HC measurements. The FAPD/OFD ratio has not been used to assess fetal frontal lobe development in fetuses with FGR. We also established the Z-score reference range of FAPD and varying pattern of the FAPD/OFD ratio in appropriately grown fetuses to evaluate the fetal frontal lobe in other diseases that may impair its development.

This study has several limitations. First, we did not evaluate neurodevelopment in infants with FGR. Second, a longitudinal design with repeated measurements, which may facilitate elucidation of FAPD/OFD ratio variations in fetuses with FGR, was not performed. We think we may benefit from a longitudinal analysis with different sampling moments to assess the changes of FAPD/OFD ratio. Finally, there are other societies and experts, such as the ACOG [35], suggest that FGR is defined as an EFW below the 10^th^ percentile. Furthermore, we have to admit that we cannot discriminate between SGA and FGR in this study.

## Conclusions

In conclusion, this study provides a marker, FAPD/OFD ratio, for evaluating fetal frontal lobe development in appropriately grown fetuses and fetuses with FGR. Fetuses with FGR showed a markedly lower FAPD Z-score and FAPD/OFD ratio than appropriately grown fetuses. Furthermore, FCR was not associated with frontal lobe development.

## Data Availability

The datasets used and/or analyzed during the current study are available from the corresponding author on reasonable request.

## Abbreviations

FGR: Fetal growth restriction
FCR: Fetal circulatory redistribution
FAPD: Frontal antero-posterior diameter
OFD: Occipito-frontal diameter
MRI: Magnetic resonance imaging
AGA: Appropriate for gestational age
BPD: Biparietal diameter
HC: Head circumference
AC: Abdominal circumference
FL: Femur length
HL: Humerus length
TCD: Transverse cerebellar diameter;
EFW: Estimated fetal weight
CSP: Cavum septum pellucidum
MCA: Middle cerebral artery
UA: Umbilical artery
UtA: Uterine artery
CPR: Cerebroplacental ratio
BMI: Body mass index
ICC: Intraclass correlation coefficient
SDs: Standard deviations
SEs: Standard errors
